# Fresh Food Consumption Increases Microbiome Diversity and Promotes Changes in Bacteria Composition on the Skin of Pet Dogs Compared to Dry Foods

**DOI:** 10.3390/ani12151881

**Published:** 2022-07-22

**Authors:** Kennedy Leverett, Rodrigo Manjarín, Erica Laird, Diana Valtierra, Tasha M. Santiago-Rodriguez, Renan Donadelli, Gerardo Perez-Camargo

**Affiliations:** 1 Carlson College of Veterinary Medicine, Oregon State University, Corvallis, OR 97331, USA; kennedyl@alumni.princeton.edu; 2 Animal Science Department, California Polytechnic State University, San Luis Obispo, CA 93407, USA; rmanjari@calpoly.edu; 3 Freshpet, Bethlehem, PA 18017, USA; elaird@freshpet.com (E.L.); dvaltierra@freshpet.com (D.V.); rdonadelli@freshpet.com (R.D.); 4 Diversigen, Inc., Houston, TX 77046, USA; tsantiagoro@gmail.com

**Keywords:** canine, microbiome diversity, pet food, skin bacteria populations

## Abstract

**Simple Summary:**

Dog skin is the first defense against the environment. There are several bacteria that live on the skin and there are differences in their types and quantities depending on the dog. Food is known to influence the bacteria in the intestine and the skin fat composition; however, it is not known if diet can impact the bacteria on the skin. The objective of this study was to evaluate if diet can change the bacteria on the skin of healthy dogs. Results from this study showed that there was an increase in bacterium types and a change in their relative quantity when dogs were fed a fresh dog food compared to these same dogs fed dry pet foods. This study was the first of its kind and shone some light on how different pet foods impact skin microbiome.

**Abstract:**

The skin is the first barrier the body has to protect itself from the environment. There are several bacteria that populate the skin, and their composition may change throughout the dog’s life due to several factors, such as environmental changes and diseases. The objective of this research was to determine the skin microbiome changes due to a change in diet on healthy pet dogs. Healthy client-owned dogs (8) were fed a fresh diet for 30 days then dry foods for another 30 days after a 4-day transition period. Skin bacterial population samples were collected after each 30-day feeding period and compared to determine microbiome diversity. Alpha diversity was higher when dogs were fed the fresh diet compared to the dry foods. Additionally, feeding fresh food to dogs increased the proportion of *Staphylococcus* and decreased *Porphyromonas* and *Corynebacterium*. In conclusion, changing from fresh diet to dry foods promoted a relative decrease in skin microbiome in healthy pet dogs.

## 1. Introduction

The skin is the first barrier that animals have to defend themselves. In dogs, diseases like pruritus, ulcers, pustules, and allergies commonly infect and affect the skin [1,2,3,4,5,6]. According to the Veterinary Practice News, in 2018 the average costs of treatment for atopic dermatitis (first most common medical condition in dogs), benign skin neoplasia (third most common), and pyoderma (fourth most common) were $255.00 USD, $377.00 USD, and $128.00 USD, respectively [7]. The percentage of the population of dogs that suffer from a skin condition is not known. However, there are pet food companies producing dog foods that aim to aid in skin health. While in most of the cases the role the microbiome plays in the severity of such diseases is not known, Chermprapai and others [8] reported an increase in the relative abundance of *Staphylococcus*, *Psychrobacter*, *Trichococcus*, and *Brachybacterium* in dogs with atopic dermatitis compared to healthy dogs. Moreover, DeCandia and co-workers [9] reported changes in skin microbiome of canids (coyotes, red foxes, and gray foxes) when infected by mites (*Sarcoptes scabiei*), in addition to facilitating secondary bacterial infections such as *Staphylococcus pseudintermedius* and *Corynebacterium*.

Aside from disease influencing microbiome, diet can change the dog skin fat composition [10,11,12] and lead to a change in bacterial populations as proposed by some research with humans [13,14]. The usual targeted nutrients are fats and specific fatty acids. For example, feeding high levels of eicosapentaenoic acid improved pruritus, alopecia, and coat character compared to corn oil [15]. Similarly, when the dog food n-6:n-3 ratio was 1:1, serum prostaglandin E3 (a proinflammatory eicosanoid from the metabolism of arachidonic acid) concentration was decreased compared to baseline levels [16]. In addition, the authors also reported an improvement in skin pruritus clinical scores [16]. Moreover, there are commercial pet foods and supplements made specifically for skin (e.g., sensitive skin, healthy skin and coat, skin, and coat care). However, to the authors’ knowledge, there are no studies relating these dietary interventions and changes in skin microbiome.

Conversely, it is well understood how nutrition can change the intestinal bacteria community [17,18,19], as the gastrointestinal tract (and its microbiome) has access to the nutrients in the food before other organs. Fibers are the most common nutrient of interest since they are not digested and absorbed by animals. Additionally, some novel research in humans indicates a relationship between the gut and the skin [20]. Thus, diet could also indirectly influence skin through changes in the gut microbiome. While dietary supplements have been evaluated in dogs with skin issues [15,21], the effects of dietary interventions in the microbiome of healthy dogs are yet to be investigated. Moreover, the effects of different food types on dog skin microbiome have not been evaluated to date. While complete and balanced diets in the US must comply with the Association of American Feed Control Officials guidelines [22], it does not mean that they are made from the same ingredients and manufactured through similar processes. Compared to the commonly known dry foods, fresh foods are processed at relatively lower temperatures and pressures. They also have a higher proportion of fresh meats, which increases the proportions of protein and fat in the food. Finally, fresh foods must be refrigerated after processing to prevent spoilage. Conversely, dry foods have higher proportions of carbohydrates and lower fat contents. Moreover, the drying process allows these diets to be shelf stable. Thus, a dry pet food will be made from different ingredients and through different processes than a fresh diet. As a result, there are differences in the nutrient profile of the diets and the nutrient availability to the animal [23], which could in turn influence the nourishment of the animal.

When considering the research with dogs, the most common practice is the use of kennel dogs to control variability of the environment and animals. However, kennel conditions are different from house conditions and kennels have a limited number of breeds used in their research. Since the environment is one of the key components known to impact the microbiome [24], the ecological validity of microbiome research using kennel dogs may be questioned when considering pet dogs. Therefore, the objective of this research was to determine the effects of fresh vs. dry diet in the skin microbiome of healthy pet dogs. The change from fresh dog food to dry diets decreased the skin microbiome diversity of healthy pet dogs tested in this work.

## 2. Materials and Methods

### 2.1. Dog Recruitment and General Study Guidelines

This study was carried out in strict accordance with the recommendations in the Guide for the Care and Use of Laboratory Animals of the National Institute of Health. All dogs enrolled in the trial were the property of responsible adults. Eight pet dogs were recruited from Bethlehem, PA, USA and Flemington, NJ, USA. The research protocol was explained to dog owners, and their conscious consent was required in the form of a formal email to K.L. for the inclusion of their animals in the trial. Dog owners were aware that they could remove their pets from the trial at any time if they desired to do so. The demographics of the dogs are reported in Table 1. Treatment with systemic antifungals or antibiotics three months prior to the start of the study was used as an exclusion criterion. All dogs were castrated. Each dog owner was instructed to (1) feed the animals according to diet manufacturer instructions, (2) adjust food intake to maintain the dog’s body weight throughout the study, and (3) refrain from feeding table food or treats for the duration of the study. Dog treats (Dog Joy, Freshpet Inc., Secaucus, NJ, USA) were provided to all participants and they were instructed to follow the feeding guidelines as outlined on the package.

### 2.2. Feeding Protocol and Diet Nutrient and Ingredient Compositions

The trial took place between September and November 2020. All dogs were fed by their owners as instructed by E.L. and K.L. All dogs were fed the Freshpet Select Sensitive Stomach and Skin roll (FPS, steam cooked for approximately 15 min at 100 °C) for 30 consecutive days, followed by their regular dry food diet (DRY, extruded) for an additional 30 consecutive days. A 4-day transition period was implemented to switch diets, during which 25% of FPS was replaced by DRY each day. FPS and DRY food samples were collected from each participant and sent to a commercial laboratory (Midwest Laboratories, Omaha, NE, USA) for analysis of moisture (AOAC 930.15), crude protein (AOAC 990.03), acid hydrolyzed fat (modified AOAC 954.02), ash (AOAC 942.05), total dietary fiber (modified AOAC 991.43), insoluble and soluble fibers (modified AOAC 991.43), fatty acid profile (AOAC 996.06), and zinc (modified AOAC 985.01). The analyzed proximate nutrient composition is presented in Table 2, selected nutrients known to impact skin health are presented in Table 3, and the list of ingredients of each diet in descending order of inclusion is provided in Supplemental Material.

### 2.3. Skin Microbiome Sample Collection

To measure changes in skin bacterial populations, swab samples from the internal ear, interdigital area of the front paw, and the groin area were collected on each dog following the 30-day feeding of FPS and DRY diets. During both sample collections dog owners were present, and they observed the procedure throughout the duration of the sampling. Samples were taken on the right side of the animals using a sterile BBL™ CultureSwab™ (BD Corporate, Franklin Lakes, NJ, USA) by rubbing the swab on each sampling site 40 times and rotating the swab by one quarter every 10 strokes [4]. Immediately after collection, samples were placed on ice and transported to Freshpet (Bethlehem, PA, USA), where they were frozen and stored at −20 °C until DNA extraction. Dog owners were asked to refrain from bathing or deodorizing the animals from 1 week before each swabbing period. To decrease the contamination with foreign bacteria, the person collecting the samples visually inspected the area to ensure that the area selected for collection was not recently licked by the dog or had any material that was not skin and hair.

### 2.4. DNA Extraction and Analysis

DNA extraction was carried out at the University of Arkansas for Medical Sciences (Little Rock, AR, USA). DNA was extracted using DNeasy^®^ PowerSoil^®^ Kit Quick-Start (Qiagen, Hilden, Germany) according to manufacturer instructions. Samples were amplified and the 16S rRNA hypervariable 4 (V4) region was tagged through PCR using 96 unique combinations of 8 forward and 12 reverse barcoded primers, as previously described [25]. PCR cycling settings were the following: initial denaturation at 94 °C for 3 min; touchdown cycling for 30 cycles of 94 °C for 45 s, 80–50 °C for 60 s, 72 °C for 90 s, decreasing 1 °C each cycle; 12 cycles of 94 °C for 45 s, 50 °C for 60 s, 72 °C for 90 s; and a final extension of 72 °C for 10 min. PCR products were quantified with Quant-iT™ PicoGreen™ dsDNA (Invitrogen, Carlsbad, CA, USA). Equal nanograms of each library were pooled, and amplicons of approximately 300 nt in length were selected using Agencourt AMPure XP magnetic beads (Beckman Coulter, Brea, CA, USA). Final libraries were sent to Princeton University Genomics Core Facility (Princeton, NJ, USA) for paired-end amplicon sequencing (2 × 150 nt) on an Illumina MiSeq machine (Illumina, San Diego, CA, SA).

### 2.5. Data Processing and Taxonomic Composition

Raw data was demultiplexed in Princeton University’s High Throughput Sequencing Database using a paired-end, dual-indexed barcode splitter that allowed one nucleotide mismatch between expected and observed barcode sequences. Demultiplexed reads were imported into QIIME2 version 2021.4 [26], and the dada2 denoise-paired function [27] was used to correct sequencing errors and combine paired-end reads for taxonomic feature identification. A rooted tree of taxonomic features was created using the QIIME2 function fasttree [28], then composition was determined, and taxonomy was assigned with the q2-feature-classifier plugin [29] and a classify-sklearn naïve Bayes classifier pre-trained on Greengenes 13_8 [30]. This classifier clustered samples at 99% similarity and trimmed sequences to only include 250 bases from the 16S V4 region. Microbiome alpha diversity analyses were performed using the core diversity_analyses.py script in the Quantitative Insights Into Microbial Ecology (QIIME1) [24] to determine observed operational taxonomic units (OTUs), Shannon, and Chao1 indexes.

### 2.6. Statistical Methods

To investigate taxa contribution to overall differences between groups, raw counts at the genus level were imported into Primer-e software (Albany, New Zealand), standardized to relative composition (so sample totals are 100%), square root-transformed, and analyzed with a Bray-Curtis similarity distance matrix. A non-parametric permutational analysis of variance (PERMANOVA; Primer-e) was used for testing the null hypothesis of no difference between groups under a reduced model, 9999 permutations, and type III sum of squares [31]. An analysis of similarity percentages (SIMPER; Primer) with a cut-off value of 70% was used to select genera contributing to the overall microbiome dissimilarity between FPS and DRY [31]. Diet-induced changes in genera selected by SIMPER were further analyzed with %Polynova SAS Macro [32]. Data are presented as fold change by DRY compared to FPS. The average values of FPS and DRY fed dogs were considered significant when *p* < 0.05.

## 3. Results

Dog D013 was excluded from the analysis due to surgery and postoperative antibiotic treatment during the study.

Nutrient composition on a dry matter basis of dry pet foods and FPS is reported on Table 2 and Table 3. The FPS diet had a greater moisture content than DRY. Moreover, on average, FPS had a higher protein and fat content and a lower carbohydrate concentration than DRY, whereas the ash content was similar. While total dietary fiber was similar among diets, the soluble fiber content of FPS was between 2.1 and 3.8 times higher than the DRY foods; consequently, the insoluble fiber content of FPS was lower than DRY diets (Table 2). Zinc content was higher in DRY foods than FPS; however, FPS had a higher concentration of linoleic, arachidonic, and DHA acids (Table 3). The FPS diet had a higher concentration of saturated and polyunsaturated fatty acids; however, the n-3 fatty acid concentration was on average lower than the DRY foods (Table 3).

**Figure 1:**
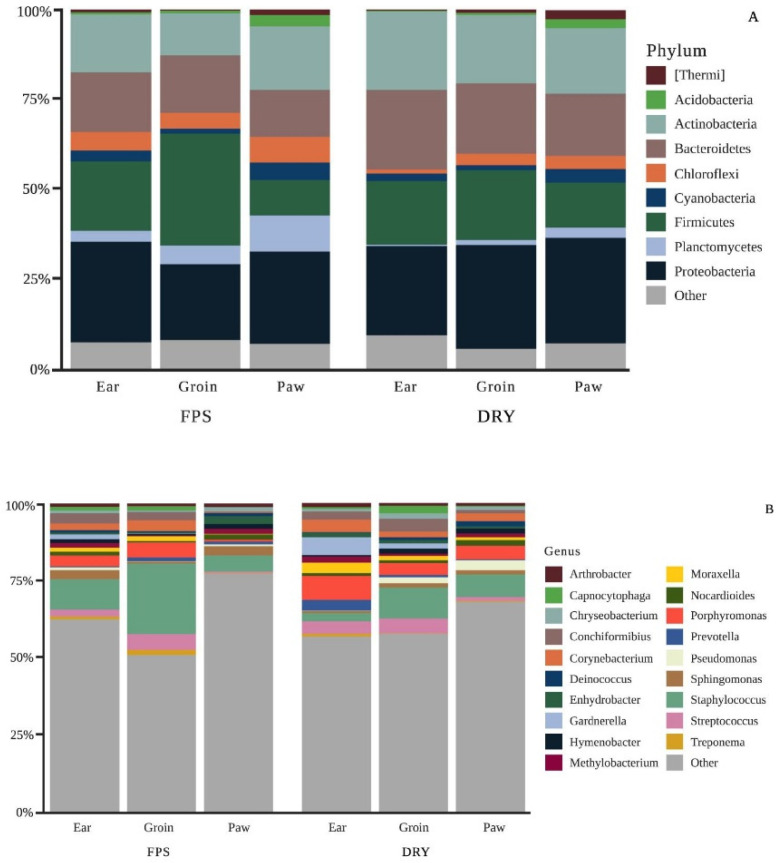
Taxonomic composition changes between body sites and diets. (**A**) Change of taxonomic composition of the skin microbial phyla and (**B**) genera for each body site between fresh (FPS) and dry (DRY) dog foods. Figures created with BioRender.com (Accessed on 15 February 2022). Supplementary Table S1 shows the relative percentages for each reported parameter.

Analysis of 16S rRNA in groin identified a total of 627 OTUs that were aggregated into 39 phyla and 421 genera. Similarly, ear analysis yielded 663 OTUs separated into 37 phyla and 435 genera, whereas in the paw 477 OTUS were aggregated into 32 phyla and 322 genera. Taxonomic composition bar plots were constructed to show the overall change of the relative percentage of phyla and genera (Figure 1) for each body site between FPS and DRY.

At the phyla level, DRY increased *Actinobacteria*, *Bacteroidetes*, and *Proteobacteria* and decreased *Planctomycetes, Firmicutes,* and *Chloroflexi* populations compared to FPS (*p* < 0.05; Figure 1A). When considering the 3 different skin sites, the groin had a higher population of *Firmicutes* compared to the other sites and the paw had higher proportions of *Cyanobacteria* and *Planctomycetes* (*p* < 0.05). The most common genera among the different skin sites and diets were *Staphylococcus*, followed by *Porphyromonas*, *Streptococcus*, *Corynebacterium*, and *Conchiformibius* (Figure 1B). The groin had higher proportions of *Staphylococcus* compared to the ear and paw (*p* < 0.05).

**Figure 2:**
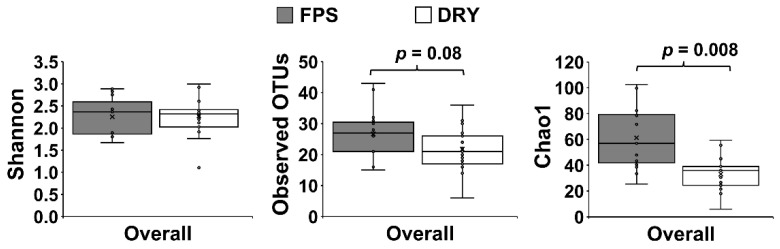
Skin microbiome alpha diversity of pet dogs fed fresh (FPS) versus dry (DRY) food. Observed OTUs, Shannon, and Chao1 indices of alpha diversity at genus level in dogs fed FPS for 30 consecutive days, followed by DRY for an additional 30 consecutive days. Values were computed using the core_diversity_analyses.py script in Quantitative Insights into Microbial Ecology. Group differences were assessed by non-parametric permutational analysis of variance with protocol and time as fixed effects, under a reduced model, 9999 permutations, and type III sum of squares.

There was an overall effect of diet in skin microbiome regardless of the area sampled (PERMANOVA *p* ≤ 0.05), with a significant decrease of alpha diversity index Chao1 in DRY compared to FPS (*p* ≤ 0.05; Figure 2).

**Figure 3:**
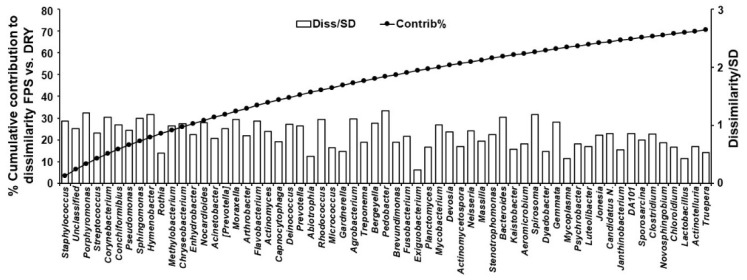
Cumulative genera percent dissimilarity of skin microbiome of pet dogs fed fresh (FPS) vs. dry (DRY) food. Cumulative % contribution of genera to dissimilarity between FPS and DRY (left Y axis), and their corresponding ratio of average dissimilarity to its standard deviation (right X axis).

Data from different swab areas were combined for further analyses. SIMPER analysis yielded a 60.41% average dissimilarity between FPS and DRY, with 61 genera contributing to 70% of differences between FPS and DRY (Figure 3).

**Figure 4:**
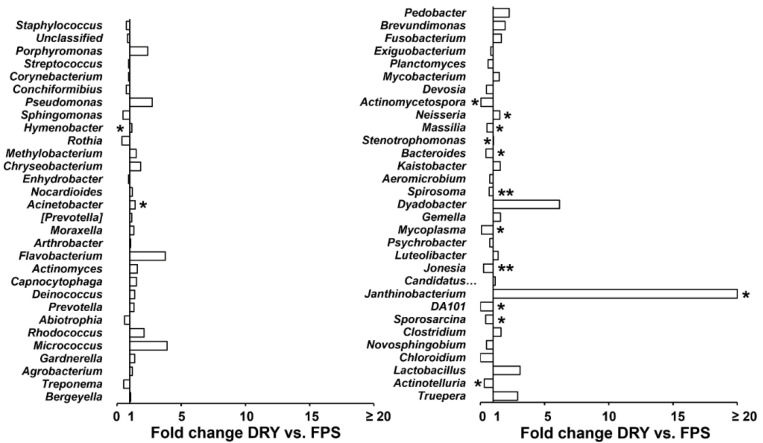
Average percentage relative counts of genera selected by SIMPER expressed as fold change. Dogs were fed FPS for 30 consecutive days, followed by their DRY for an additional 30 consecutive days. Data were analyzed by a one-way ANOVA that included diet as fixed effect. * *p* ≤ 0.05, ** *p* ≤ 0.01.

*Staphylococcus*, *Porphyromonas*, *Streptococcus*, *Corynebacterium*, *Conchiformibius*, and *Pseudomonas* showed the largest percentage contribution to dissimilarity between FPS and DRY, whereas *Pedobacter*, *Porphyromonas*, *Hymenobacter*, *Spirosoma*, *Corynebacterium*, and *Bacteroides* were the best discriminators, as their ratio of average dissimilarity to its standard deviation were the highest (Figure 3). Compared with FPS, DRY increased (*p* ≤ 0.05) average percentage relative counts of *Hymenobacter*, *Acinetobacter*, *Neisseria*, *Stenotrophomonas,* and *Janthinobacterium*, and decreased (*p* ≤ 0.05) *Actinomycetospora, Massilia, Bacteroides, Spirosoma, Mycoplasma, Jonesia, DA101, Sporosarcina,* and *Actinotelluria* (Figure 4).

## 4. Discussion

The objective of this study was to investigate whether diet (fresh vs. dry food) would have an impact on the skin microbiome of pet dogs. Because of the differences in the environment that pet vs. kennel dogs are subjected to and the effects of the environment on microbiome, the results here presented are an attempt to generate data that would represent the housing conditions that most of the dogs in the US live in. Case in point, when the nasal and oral microbiome of detection dogs of different locations were tested, there was a difference in nasal Chaos1 diversity of dogs housed in different states in the United States [33]. While the number of animals enrolled was a limitation of this work, the variability in the dry foods consumed by the selected pet dogs was chosen as an attempt to mimic a real-life situation in which dog owners decided to change dog foods. No statistical analyses were performed among the different dry foods to investigate if they would have an influence in skin microbiome, as this was not the goal of this research and there were not enough experimental units for such analyses. The dietary differences go beyond the nutrient composition, but also the ingredient content, particularly the protein and the fat sources of the diets. These differences in ingredients and processing methods likely affected the nutrient availability of the diets. For example, the amino acid availability of different chicken-based protein changed depending on how these proteins were processed [23]. For the health of the skin, perhaps the fat of the diet might have a greater impact than the protein, since the outer layer of the epidermis, the stratum corneum, is mainly composed of different fat compounds [14]. Overall, FPS have a higher fat content than DRY foods, thus this may have better supported the health of the skin. Of all the fatty acids, linoleic acid has a key role in the formation of the stratum corneum, as it is bound to cornified envelopes to create a scaffolding structure for free ceramides present among the corneocytes [14]. The formation of these bonds is essential for the “brick and mortar” structure of the stratum corneum and the proper barrier function that the skin has [34]. Since the fat content of the skin can select different bacteria to grow on the surface of the skin [13,14] and changes in dietary fat composition promote changes in the skin fat composition [10,11,12], perhaps when combined, these two factors might promote a change in the skin microbiome.

There are two main factors that may have supported the compositional changes in skin bacteria populations in this study: the type of the diet fed and changes in weather conditions. Because the trial was performed from September through November 2020, there were seasonal changes that might have affected the skin microbiome, since environmental conditions are a known factor that impacts skin microbiome [35,36]. However, because all dogs enrolled in the trial were indoor dogs with limited access to outdoors (occasional walks and visits to the dog park), most likely the environmental conditions had limited effects on the changes reported here, as the dogs would spend most of their time indoors under controlled conditions. Thus, the diet change might be the main factor contributing to the changes in microbial diversity, with seasonal environmental conditions playing a smaller role. In a study that evaluated the skin microbiome of healthy and allergic dogs [4], the diversity of the bacterial population was lower in allergic dogs compared to healthy dogs. However, it is not known if the change is a cause or an effect of the disease. In addition, the skin microbiome of dogs with atopic dermatitis was reported to have less diversity and higher relative concentrations of *Staphylococcus* (especially *S. pseudintermedius*) and *Corynebacterium* compared to healthy dogs [5]. Therefore, improving skin microbiome could be beneficial in preventing or waning some of these illnesses. In this study, there was a decrease in diversity when dogs were fed DRY compared to FPS. Moreover, the abundance of *Corynebacterium* was higher in dogs fed DRY; however, the abundance of *Staphylococcus* was lower compared to dogs fed FPS. Although these results are conflicting, it is important to determine the species of the bacteria on the skin, as some specific species are related to certain conditions [37]. For example, while the increased abundance of *Staphylococcus* when dogs were fed FPS could be concerning, there is the need to further investigate what species was increased. For example, in humans, *Staphylococcus epidermidis* can prevent the colonization of *Staphylococcus aureus* [38]. While this has not been proven true for dogs, it would be prudent to assume that the increase of *Staphylococcus* abundance would be detrimental for the dog’s health. From research done with human subjects, it is known that different skin sites have different concentrations of sebum and bacteria populations [13,39]. The present research reported differences in bacteria composition in the different sites analyzed; however, the skin sebum content was not evaluated, and it should be addressed in future research.

Although this study was not designed to investigate specific components in the diet, differences in nutrient composition between FPS and DRY foods may have partially contributed to the observed differences in skin microbial population. Diet nutrients (such as zinc, higher fat content, and specific fatty acids) are known to impact the composition of the skin [11,21]. Zinc is involved as a cofactor for RNA and DNA polymerases and in the activation of delta-6-desaturase, an enzyme that converts linoleic acid into arachidonic acid. Zinc concentration and availability are of particular interest for the health of the skin, due to skin’s constant cell divisions to replenish the cells lost by desquamation. There were different sources of zinc used in the experimental diets, such as zinc oxide, zinc proteinate, and zinc sulfate. While all these zinc sources are recognized by AAFCO, zinc proteinate was reported to be more bioavailable than zinc oxide and zinc sulfate [40,41]. A more available zinc source would possibly contribute to the health of the skin. As reported previously elsewhere [41], the diet with zinc proteinate increased the hair brightness of different areas of the dog’s body, which the authors considered as a healthier coat. These same authors also reported that dogs fed zinc proteinate had an immune response that persisted longer than dogs fed zinc oxide. This persistent immune response could be beneficial for dogs in controlling pathogenic bacteria colonization on the surface of the skin.

Another major difference among these diets is the soluble fiber content (Table 2). Soluble fibers are known to change the gut microbiome composition [42,43,44]. Fiber fermentation is beneficial to the host with the provision of short-chain fatty acids, specifically butyrate, which has been shown to regulate some of the host’s physiology [45]. Moreover, there is novel research showing a relationship between the gut and skin [20,36]. While the mechanisms behind the gut–skin axis are not well understood to date, the gut microbiome can indirectly influence the skin microbiome by modulating the host immune system. Due to the novelty of gut–skin axis, even for human research, any associations with a dog model must be approached carefully, although it would be safe to assume that the higher proportions of soluble fibers in FPS would stimulate fermentation and bacterial growth in the colon when dogs were fed this diet. This increase in bacteria populations and production of fermentation products could stimulate the immune system and promote changes on how the skin would react to different bacteria. However, neither the gut microbiome nor the fermentation products were measured in the feces of the dogs enrolled in the trial. Since the gut bacteria can modulate the immune system, it would be prudent to consider different measurements of the immune system in future studies.

Finally, the water content of FPS was much higher than the DRY foods. However, dogs are known to regulate water intake regardless of the amount of water present in the food to maintain water balance [46,47]. Furthermore, the evaluation of the water balance was not the intent this study. In humans it was reported that drinking 1 L more than their baseline water intake [48] improved skin hydration status; however, the microbiome of the skin was not evaluated. Moreover, Mukherjee and co-workers [49] reported that there are differences in bacterial distribution on the face of women depending on the sebum or hydration of the skin, although it is necessary to mention that the water intake was not evaluated in that publication [49]. It is unknown if this is relevant for dogs, since humans can willingly increase water intake to a certain level to meet research protocols and dogs most likely would not voluntarily drink more.

As mentioned previously, the preset study has limitations. The small number of animals, the use of different dry pet foods, and the change of environmental conditions as the trial progressed are the main limitations. Future studies should take these into consideration when designing new trials.

## 5. Conclusions

In conclusion, changing FPS diet to DRY promoted a decrease in skin microbiome relative abundance in dogs. Nevertheless, future research should evaluate the water balance, the colonic microbiome, and the immune system when analyzing the skin microbiome, since these factors may have an impact the skin bacteria populations.

## Figures and Tables

**Table 1:** Demographic information of dogs enrolled in the study.

Dog ID	Breed	Sex	Age (Years)
D010	Mixed	Male	1
D011	Goldendoodle	Male	3
D012	Australian Cattle	Male	8
D013	Mixed	Female	2
D014	Mixed	Male	0.8
D015	Mixed	Male	5
D016	American Bully	Male	5
D017	American Bully	Female	4

**Table 2:** Moisture and macro nutrient content on dry matter basis of the experimental diets (%), metabolizable energy data (kcal/kg diet), and zinc (mg/kg of diet on a dry matter basis).

Nutrient	DRY 1	DRY 2	DRY 3	DRY 4	DRY 5	DRY Average	FPS
Moisture	7.9	9.9	7.6	10.4	6.4	8.5	76.3
Crude Protein	37.2	33.8	40	26.8	28.4	33.0	43.9
Fat	20.5	11.8	19.7	15.4	13.2	16.1	21.4
Ash	10.1	7.8	9.6	7.2	5.7	8.1	7.9
Total Dietary Fiber	12.2	10.9	12.0	8.0	14.2	11.5	11.8
Insoluble Fiber	9.8	8.9	9.4	6.6	12.1	9.4	6.3
Soluble Fiber	2.4	2.0	2.6	1.5	2.1	2.1	5.5
Nitrogen-free Extract ^1^	19.4	35.8	19.8	42.5	38.5	31.2	17.7
Metabolizable Energy ^2^, Dry Basis (Wet Basis)	3724 (3429)	3439 (3099)	3768 (3481)	3735 (3346)	3464 (3242)	3616 (3308)	3975 (942)
Zinc	265	289	232	208	222	243	215

^1^ Nitrogen-free extract = 100-Moisture-Crude protein-Fat-Ash-Total dietary fiber. ^2^ Metabolizable Energy estimated based on the Modified Atwater values of 3.5 for crude protein and nitrogen-free extract and 8.5 for fat, as outlined by the AAFCO.

**Table 3:** Selected fatty acids content (% of dry matter) of experimental diets.

Nutrient	DRY 1	DRY 2	DRY 3	DRY 4	DRY 5	DRY Average	FPS
Saturated fats	6.58	2.71	5.78	6.63	3.29	5.00	6.82
Monosaturated fats	8.41	4.64	8.49	5.84	4.72	6.42	8.85
Polyunsaturated fats	3.94	3.18	3.87	1.28	4.22	3.30	5.21
ω-6 fatty acids	3.65	2.50	3.20	1.18	3.35	2.78	4.78
ω-3 fatty acids	0.26	0.67	0.63	0.10	0.85	0.50	0.39
ω-6: ω-3 ratio ^1^	14.04	3.73	5.08	11.80	3.94	5.56	12.26
Linoleic acid	3.51	2.38	3.06	1.14	3.26	2.67	4.32
α-Linolenic acid	0.23	0.42	0.44	0.09	0.82	0.40	0.24
Arachidonic acid	0.08	0.06	0.08	0.02	0.04	0.06	0.35
Eicosapentaenoic acid	nd ^2^	0.07	0.09	nd	nd	0.03 ^1^	0.03
Docosahexaenoic acid	nd	0.14	0.08	nd	nd	0.04 ^1^	0.09

^1^ ω-6: ω-3 ratio calculated as ω-6 fatty acids divided by ω-3 fatty acids. ^2^ nd considered in the average value as zero.

## Data Availability

The data presented in this study are available on request from the corresponding author.

## Supplementary Materials

The following supporting information can be downloaded at: www.mdpi.com/article/10.3390/ani12151881/s1, Table S1: Relative percentages of the most common Phyla and genera of dogs fed FPS and DRY foods.
