# Supplementary material for: Fresh Food Consumption Increases Microbiome Diversity and Promotes Changes in Bacteria Composition on the Skin of Pet Dogs Compared to Dry Foods
Source: Animals (Basel). 2022 Jul 22;12(15):1881. doi: 10.3390/ani12151881 (PMC9329806; doi:10.3390/ani12151881)
Supplement: Supplementary file 1 [file animals-12-01881-s001.zip › animals-1619832-supplementary.pdf]

Table S1. Relative percentages of the most common Phyla and genera of dogs fed FPS and DRY foods.

	FPS			DRY		
	**Ear**	**Groin**	**Paw**	**Ear**	**Groin**	**Paw**
				**Phyla**		
[Thermi]	0.65	0.31	1.46	0.24	0.85	2.69
Acidobacteria	0.60	0.72	3.16	0.35	0.56	2.43
Actinobacteria	16.14	11.65	17.62	21.74	18.99	18.25
Bacteroidetes	16.67	15.98	13.09	22.09	19.68	17.29
Chloroflexi	5.07	4.43	7.19	1.16	3.16	3.65
Cyanobacteria	3.12	1.41	4.91	2.03	1.35	3.81
Firmicutes	19.19	31.08	9.86	17.69	19.54	12.38
Planctomyces	3.02	5.24	9.90	0.46	1.26	2.91
Proteobacteria	28.00	21.03	25.79	24.79	28.90	29.36
Other	7.54	8.15	7.02	9.46	5.72	7.21
				**Genera**		
Arthrobacter	0.97	0.83	1.08	1.24	0.91	0.86
Capnocytophaga	1.24	1.44	0.05	0.73	2.36	0.22
Chryseobacterium	0.91	0.34	1.38	0.71	1.87	1.10
Conchiformibius	3.43	2.79	0.20	2.77	4.06	1.06
Corynebacterium	2.12	3.51	0.33	3.93	1.90	2.56
Deinococcus	0.63	0.31	0.98	0.21	0.85	1.68
Enhydrobacter	0.60	0.48	2.67	1.51	1.00	0.64
Gardnerella	1.61	0.12	0.00	5.68	1.77	0.03
Hymenobacter	1.22	0.45	1.40	0.53	1.70	1.60
Methylobacterium	1.44	0.25	1.77	1.95	0.75	1.30
Moraxella	1.42	1.53	0.14	3.31	1.28	0.82
Nocardioides	1.25	0.57	1.62	0.90	0.92	1.83
Porphyromonas	3.40	4.78	0.58	7.85	3.88	4.42
Prevotella	0.43	1.30	0.96	3.55	0.70	0.30
Pseudomonas	0.90	0.13	0.54	0.19	2.01	3.21
Sphingomonas	2.80	0.63	3.09	0.58	1.31	1.39
Staphylococcus	10.11	22.91	5.21	2.83	10.17	7.36
Streptococcus	2.25	5.15	0.44	3.86	4.74	1.14
Treponema	0.93	1.59	0.11	0.94	0.22	0.29
Other	62.33	50.88	77.43	56.74	57.60	68.19
